# Enhancement of docetaxel-induced cytotoxicity and apoptosis by all-trans retinoic acid (ATRA) through downregulation of survivin (BIRC5), MCL-1 and LTbeta-R in hormone- and drug resistant prostate cancer cell line, DU-145

**DOI:** 10.1186/1756-9966-27-37

**Published:** 2008-09-12

**Authors:** Yuksel Kucukzeybek, Mustafa K Gul, Ercument Cengiz, Cigdem Erten, Burcak Karaca, Gurbuz Gorumlu, Harika Atmaca, Selim Uzunoglu, Bulent Karabulut, Ulus A Sanli, Ruchan Uslu

**Affiliations:** 1 Division of Medical Oncology, Tulay Aktas Oncology Hospital, School of Medicine, Ege University, Bornova, Izmir, Turkey; 2 Section of Molecular Biology, Department of Biology, Faculty of Science and Arts, Celal Bayar University, Muradiye, Manisa, Turkey

## Abstract

**Background:**

The management of hormone-refractory prostate cancer (HRPC) still remains as an important challenge of daily oncology practice. Docetaxel has proved to be a first line treatment choice. All-trans retinoic acid (ATRA) could potently inhibit the growth of prostate cancer cells *in vitro *and its combination with various anticancer agents results in increased cytotoxicity. Based on these data, our aim was to examine the synergistic/additive cytotoxic and apoptotic effects of combination of docetaxel and ATRA, in hormone- and drug refractory human DU-145 prostate cancer cells. Furthermore, we have searched for the underlying mechanisms of apoptosis by demonstrating apoptosis-related genes.

**Methods:**

XTT cell proliferation assay was used for showing cytotoxicity. For verifying apoptosis, both DNA Fragmentation by ELISA assay and caspase 3/7 activity measurement were used. For detecting the mechanism of apoptosis induced by docetaxel-ATRA combination, OligoGeArray^® ^which consists of 112 apoptosis related genes was used.

**Results:**

Our results revealed that docetaxel and ATRA were synergistically cytotoxic and apoptotic in DU-145 cells, in a dose- and time dependent manner. It was also shown by our studies that apoptosis was induced in DU-145 prostate carcinoma cells with significant cytotoxicity, no matter which agent applied first. We have found out that docetaxel-ATRA combination significantly downregulates survivin (BIRC5), myeloid cell leukemia-1 (MCL-1) and lymphotoxin β-receptor (LTβR) genes, which all three have pivotal roles in regulation of apoptosis and cell cycle progression.

**Conclusion:**

In conclusion, we strongly suggest that docetaxel and ATRA combination is a good candidate for this challenging era of daily oncologic practice. Also, the combination of docetaxel and ATRA might allow a reduction in docetaxel doses and by this way may diminish docetaxel adverse effects while maintaining the therapeutic effect in patients with HRPC.

## Background

Prostate cancer is the second most common cause of cancer death among men in developed countries [1]. In about 80% of metastatic prostate cancer patients, primary androgen ablation leads to symptomatic improvement and a decrease in serum prostate-specific antigen (PSA), but in all patients the disease will eventually become refractory to hormone treatment [2]. These patients have a median survival of 16 to 18 months, despite modern chemotherapy.

Docetaxel is a semisynthetic anticancer agent which shows its activity through stabilizing microtubules during cell division. In addition to this cytostatic ability, it can also regulate cell signaling and expression of certain genes. Docetaxel treatment increases Bcl-2 phosphorylation, downregulates Bcl-XL protein levels, induces p53 and antiangiogenic factors and thus results in apoptosis [3]. Although docetaxel chemotherapy has become the first-line standard of care for HRPC based on the results of two large randomized trials, PSA responses rarely exceed 50% and median survival is less than 20 months, thus the use of chemotherapy in this disease remains a subject of active clinical investigation [4]. There are also some problems encountered during docetaxel treatment including serious side effects in most of the patients [3,5,6]. Investigators are now focused on how to enhance the cytostatic and cytotoxic effects of docetaxel by combining it with novel anticancer agents for the treatment of prostate cancer.

All-trans retinoic acid (ATRA) is a natural analog of retinoic acids which can regulate a number of cellular activities including proliferation, differentiation, metabolism, reproduction, morphogenesis, and induction of apoptosis. ATRA shows most of its effect by binding specific nuclear retinoic acid receptors (RARs). Since ATRA can regulate aberrant cell growth and induce apoptosis, it has been widely investigated in preclinical and clinical trials to be used in the treatment of many cancer types, including prostate cancer [7-11]. It was also demonstrated by different groups that ATRA can be used to increase the sensitivity of different types of cancers to applied anticancer agents [12-15].

Based on these data, we examined the synergistic/additive effects of combination of docetaxel and the natural analogue of retinoic acids, ATRA, in hormone- and drug refractory human DU-145 prostate cancer cells. These cells are ideal models to study the effects and mechanisms of various anticancer agents since they represent very high aggressive nature of metastatic human prostate cancers. We also studied the possible apoptotic mechanisms induced by combination of both and any agent alone in these cells.

## Materials and methods

### Chemicals

Cell culture supplies were obtained from Biological Industries (Kibbutz Beit Haemek, Israel). Docetaxel and ATRA were obtained from Sigma Chemical Co (USA). The stock solution of ATRA was prepared at a concentration of 83 mM in DMSO and aliquots were stored at -20°C. Stock solution of docetaxel (10 mM) was prepared in DMSO and the DMSO concentration in the assay did not exceed 0,1%. All the other chemicals, unless mentioned, were obtained from Sigma (USA).

### Cell lines and culture conditions

Human DU-145 prostate cancer cells were obtained from ICLC (Genova, Italy). The cells were maintained in RPMI 1640 growth medium containing 10% heat-inactivated fetal bovine serum (FBS), 1% L-glutamine, and 1% penicillin-streptomycin at 37°C in 5% CO2.

### Measurement of growth by XTT cell proliferation assay

The cytotoxic effect of ATRA, docetaxel, and combination of both on DU145 cells were determined by using XTT cell proliferation assay (Roche Applied Science, Mannheim, Germany).

In short, cells (10^4 ^cells/well) were plated into 96-well plates containing 200 μl of the growth medium in the absence or presence of increasing concentrations of the anticancer agents at 37°C in 5% CO_2 _after verifying cell viability by trypan blue dye exclusion assay. After incubation period of 72 h, 50 μl of XTT labeling mixture was added to each well. Finally, the optical density was measured at 490 nm with a reference wavelength at 650 nm in a microplate reader (Beckman Coulter, DTX 880 Multimode Reader). After that, the IC_50 _concentrations of the compound were calculated from cell proliferation plots. Triplicate wells were used for each treatment.

### Measurement of DNA fragmentation by ELISA assay

DNA fragmentation levels were measured by ELISA using Cell Death Detection ELISAPLUS kit (Roche Applied Science, Mannheim, Germany). The relative amounts of mono- and oligonucleosomes generated from the apoptotic cells were quantified using monoclonal antibodies directed against DNA and histones by ELISA. Briefly, cytoplasmic fraction of the untreated controls and ATRA or docetaxel alone and in combination of both treated DU-145 cells were transferred onto a streptavidin-coated plate and were incubated at room temperature for 2 hours with a mixture of peroxidase conjugated anti-DNA and biotin labeled anti-histone. The plate was washed thoroughly and incubated with 2,29-Azino-di-[3-ethylbenzthiazolinesulfonate] diammonium salt (ABTS) before measuring the absorbance at 405 nm with a reference wavelength of 490 nm (Beckman Coulter, DTX 880 Multimode Reader). All the experiments were performed in duplicate in enzyme-linked immunosorbent assay (ELISA) assays.

### Measurement of caspase 3/7 enzyme activity

Caspase 3/7 enzyme activity was determined using the Caspase-Glo 3/7 assay (Promega, Madison, WI) as described by the manufacturer. Briefly, DU-145 cells at a concentration of 10^4 ^cells/well were plated in a 96-well plate in 100 μl culture medium in the absence or presence of increasing concentrations of ATRA or docetaxel alone and in combination of both for 72 h. Then, 100 μl of Caspase-Glo 3/7 reagent was added on to each well and the plates were incubated at room temperature for 1 more hour. Finally, the luminescence of each sample was measured at luminometer (Beckman Coulter, DTX 880 Multimode Reader).

### Examining the expression levels of apoptosis specific genes by oligoarray

Expression levels of apoptosis specific genes were examined by human apoptosis Oligo GEArray^® ^(SuperArray, Frederick, MD). The Oligo GEArray^® ^Human Apoptosis Microarray profiles the expression of 112 genes involved in apoptosis. This array includes TNF ligands and their receptors, members of the Bcl-2 gene family and caspases. Briefly, total RNA was extracted from cell samples using an Array Grade Total RNA isolation kit (SuperArray, Frederick, MD) and quantitated by UV spectroscopy using a Eppendorf biophotometer. The integrity and quality of the isolated RNA was determined by running the RNAs on agarose gel electrophoresis. cDNA was labeled from total RNA with biotin-16-dUTP and the GE Array TM Amp Labelling-LPR Kit (SuperArray, Frederick, MD) according to manufacturer instructions. The biotin-labeled cDNA was then added to the membrane and hybridized overnight to Human Apoptosis Oligo GEArray as stated by the manufacturer. Signal detection was achieved by exposure to CDP-Star alkaline phosphatase chemiluminescent substrate (SuperArray, Frederick, MD). An image was processed using Kodak Gel Logic 1500 Imaging System and analysed with the GEArray analyser software. Experiments were repeated three times using RNA extracted from three different cultures.

### Statistical analysis

All experiments were set up in triplicate and the results were expressed as the mean ± standard deviation (SD). Statistical analysis and P values determinations were conducted by the Student's *t*-test. The data were analyzed using GraphPad PRISM software (version 5, CA, USA). Median dose effect analysis was used to assess the interaction between agents. Determination of the synergistic versus additive versus antagonistic cytotoxic effects of the combination treatment of the cells by docetaxel and ATRA was assessed by Biosoft CalcuSyn program (Ferguson, MO, USA). CI was used to express synergism (CI < 1), additive effect (CI = 1), or antagonism (CI > 1) [16].

## Results

### Effects of docetaxel and ATRA on the growth of human DU-145 prostate cancer cells

**Figure 1:**
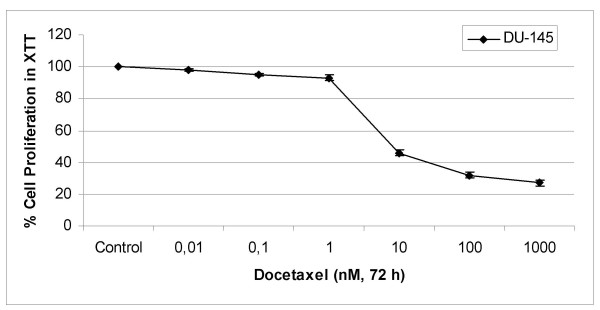
Cytotoxic Effects of Docetaxel on DU-145 cells. The XTT assays were performed using triplicate samples in at least two independent experiments. The error bars represent the standard deviations, and when not seen, they are smaller than the thickness of the lines on the graphs. *P *< 0,05 was considered significant.

To evaluate the effects of docetaxel and ATRA on the growth of human prostate cancer cells, DU-145 cells were exposed to increasing concentrations of docetaxel (from 0,01- to 1000 nM) and ATRA (from 40- to 140 μM) for 24, 48 and 72 h. Both docetaxel and ATRA decreased cell proliferation in a time- and dose dependent manner in cells (data not shown). As shown in figure 1, there were 5-, 7-, and 54% decreases in cell proliferation in 0,1-, 1-, and 10 nM docetaxel applied DU-145 cells, as comparing to untreated controls at 72 h. Highest cytotoxicity was observed at 72 h and IC_50 _value of docetaxel in DU-145 (figure 1) cells was calculated from cell proliferation plots and was found to be 8,2 nM.

**Figure 2:**
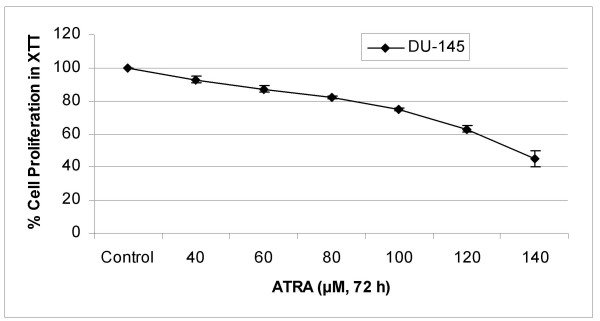
Cytotoxic Effects of ATRA on DU-145 cells. The XTT assays were performed using triplicate samples in at least two independent experiments. The error bars represent the standard deviations, and when not seen, they are smaller than the thickness of the lines on the graphs. *P *< 0,05 was considered significant.

We conducted the same set of experiments for ATRA and our results showed that in 40-, 80-, and 120 μM ATRA exposed DU-145 (figure 2) cells, there were 7-, 18-, and 37% decreases in cell proliferation, as compared to untreated controls. IC50 value of ATRA was 135 μM for DU-145 cells (figure 2).

### Exposure to combination therapy of docetaxel and ATRA resulted in a synergistic cytotoxicity as compared to any agents alone in human DU-145 prostate cancer cells

To study the possible synergistic/additive effects of docetaxel and ATRA combination, DU-145 cells were exposed to different concentrations of docetaxel or ATRA alone, and in combination of both for 24, 48 and 72 hours. The synergism or additivity was calculated via combination index (CI) by using Biosoft Calcusyn Program. CI = 1 indicates additive effect, CI > 1 indicates antagonism, CI < 1 indicates synergism and CI < 0.5 shows strong synergy. Combination of different concentrations of docetaxel and ATRA were evaluated at different time points (data not shown). Results showed significant synergistic toxicity on prostate carcinoma cells at 72 h, as compared to any agent alone as shown in table 1. The results revealed that while 0,1 nM docetaxel and 60 μM ATRA resulted in 5- and 13% decrease in proliferation of DU-145 cells, respectively, the combination of both drugs at the same doses caused 78% decrease in cell proliferation as compared to untreated controls.

**Table 1:** Combination index values of docetaxel or ATRA alone and their combination on growth inhibition of DU-145 cells.

**Concentration of drugs**	**CI value**	
DOC (0,01 nM) + ATRA (60 μM)	0.254	Strong synergism
DOC (0,1 nM) + ATRA (80 μM)	0.300	Strong synergism
DOC (0,1 nM) + ATRA (60 μM)	0.219	Strong synergism

Combination index (CI) values were calculated from the XTT cell proliferation assays. The data represent the mean + SD of 3 independent experiments.

**Figure 3:**
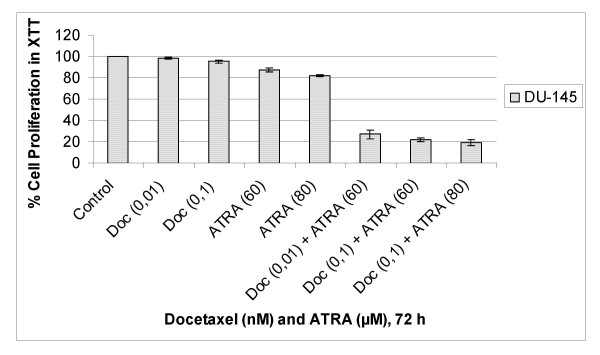
Antiproliferative Effects of Combination of Docetaxel and ATRA on DU-145 Cells. Cytotoxicity was determined by the XTT cell proliferation test in a 72 hours culture. The results are expressed as the mean of three different experiments. The error bars represent the standard deviations, and when not seen, they are smaller than the thickness of the lines on the graphs. *P *< 0,05 was considered significant.

The synergistic effect of combination of docetaxel and ATRA is best observed in their lower doses as shown in figure 3. There were strong synergism calculated for 0,01 nM docetaxel and 60 μM ATRA, 0,1 nM docetaxel and 80 μM ATRA and 0,1 nM docetaxel and 80 μM ATRA in DU-145 cells (table 1).

### Effects of the sequential treatment

The previous findings demonstrated that tumor cells with docetaxel and ATRA resulted in significant synergy at 72 h. We examined the effect of sequential treatment of DU-145 cells with either docetaxel or ATRA and subsequent treatment with the second agent. Pretreatment of tumor cells with docetaxel for 36 h and wash and then treatment for an additional 36 h with ATRA resulted in synergistic cytotoxicity in DU-145 cells. Also, pretreatment of tumor cells with ATRA for 36 h and wash and then treatment for an additional 36 h with docetaxel resulted in synergistic cytotoxicity in DU-145 cells (data not shown). So, we could not show any effect of sequential treatment on synergy obtained with either of the agents.

### Combination of docetaxel and ATRA induced DNA fragmentation significantly as compared to any agent alone in DU-145 prostate cancer cells

**Figure 4:**
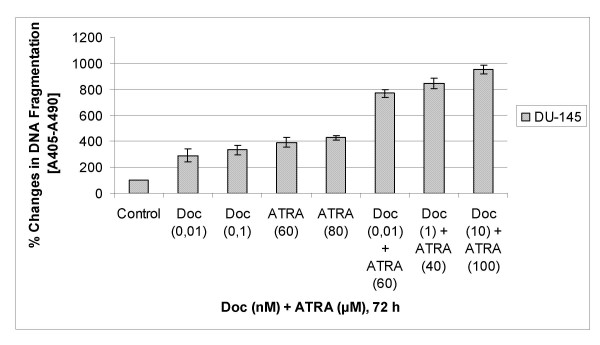
Apoptotic effects of docetaxel and ATRA alone or in combination on DU-145 cells through DNA fragmentation analyses. The results are the means of two independent experiments. The error bars represent the standard deviations, and when not seen, they are smaller than the thickness of the lines on the graphs. P < 0,05 was considered significant.

To examine the possible synergistic effects of combination of docetaxel and ATRA, as compared to any agent alone, on induction of DNA fragmentation as a marker of cell death, we quantified the levels of mono-oligo nucleosome fragments using Cell Death Detection Plus Elisa Kit (Roche Applied Science, Mannheim, Germany). We treated DU-145 cells in different concentrations of docetaxel or ATRA and the combination of both for 72 hours before analysing DNA fragmentations (figure 4). The results showed that when DU-145 cells exposed to 0,01 nM docetaxel and 60 μM ATRA, there were 2,9- and 3,9 times increase observed in DNA fragmentation as the combination of both induced DNA fragmentation 7,7 times more as compared to untreated controls (figure 4).

### A significant increase in caspase-3/7 enzyme activity has been observed in response to docetaxel and ATRA combination as compared to any agent alone in human prostate cancer cells

**Figure 5:**
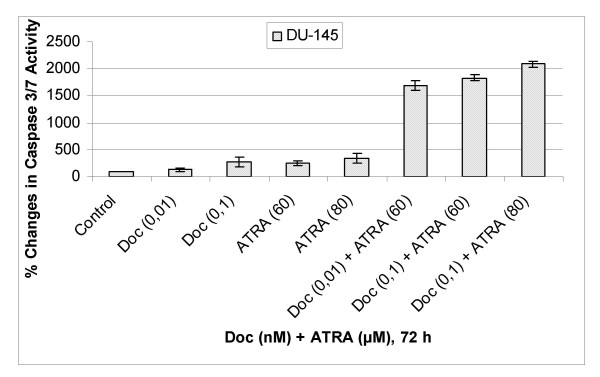
Percent Changes in Caspase 3/7 Enzyme Activity in Docetaxel or ATRA and their combination exposed DU-145 Cells. The results are the means of two independent experiments. The error bars represent the standard deviations, and when not seen, they are smaller than the thickness of the lines on the graphs. P < 0,05 was considered significant.

In order to better evaluate the possible synergistic effects of combination of docetaxel and ATRA, as compared to any agent alone, on induction of apoptosis in human DU-145 cells, we performed caspase 3/7 enzyme activity assay using Caspase-Glo 3/7 Assay (Promega, Madison, WI). To that aim, DU-145 cells were exposed various concentrations of docetaxel or ATRA alone and in combination of both for 72 h (figure 5).

In parallel with the DNA fragmentation analyses, our results revealed that there was a dose-dependent increase in caspase 3/7 activation both in docetaxel and ATRA exposed DU-145 cells. Specifically, in DU-145 cells exposed to 0,01 nM docetaxel or 60 μM ATRA, there were 1,25- and 2,5 times increases in caspase 3/7 enzyme activity, respectively, while combination of both resulted in 17 times increase in caspase 3/7 enzyme activity (figure 5) as compared to untreated controls.

### Docetaxel and ATRA combination significantly reduce mRNA levels of antiapoptotic survivin (BIRC5), MCL-1 and LTβR genes in DU-145 cells

**Figure 6:**
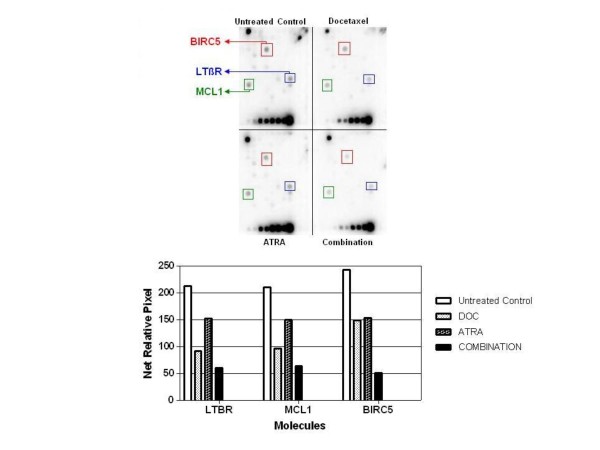
Changes in mRNA levels of apoptosis related genes in DU-145 cells exposed to docetaxel and ATRA combination as compared to untreated controls. mRNA profiles of apoptosis related genes were obtained by using Oligo GEArray Human Apoptosis (SuperArray, Frederick, MD, USA). mRNAs were isolated from untreated, docetaxel treated, ATRA treated and combination of docetaxel and ATRA treated DU-145 cells. Survivin (BIRC5), MLC-1 and LTβR mRNA levels were significantly downregulated by combination treatment. P < 0,05 was considered significant.

We used apoptosis specific oligoarray to examine the changes in expression levels of the apoptosis-related genes in response to docetaxel (0.1 nM) and ATRA (60 μM) treatment in DU-145 cells as compared to untreated controls. These doses of both docetaxel and ATRA were chosen because of best synergy obtained in cytotoxicity experiments. mRNA expression results from this study are presented by degree of significance of fold change. Three repeated experiments were carried out and the results showed that there were 3.5-, 3.3 and 4.8, fold decrease in mRNA levels of antiapoptotic survivin (BIRC5), myeloid cell leukemia-1 (MCL-1) and lymphotoxin β-receptor (LTβR) genes, respectively, in DU-145 cells exposed to combination of docetaxel and ATRA compared to untreated controls (figure 6). These were the only genes that have been significantly downregulated out of 112 apoptosis-related genes by the docetaxel and ATRA combination treatment.

## Discussion

Data presented here provide evidence that treatment of hormone- and drug resistant prostate cancer cell line, DU-145 with combination of docetaxel and ATRA results in a significant synergistic cytotoxic activity and apoptosis. The findings demonstrate that the synergistic effect of combination of docetaxel and ATRA is best observed in their lower doses which acceptable for *in vivo *administration.

We have looked for the possible underlying mechanisms of synergy achieved by combination of docetaxel and ATRA and we have shown that combination significantly downregulates survivin (BIRC5), myeloid cell leukemia-1 (MCL-1) and lymphotoxin β-receptor (LTβR) genes, which all three have pivotal roles in regulation of apoptosis and cell cycle progression. Since apoptosis and cell cycle are closely linked processes, there are a number of regulatory molecules that interface between apoptosis and cell cycle progression. In the process of carcinogenesis, altered regulation between apoptosis and cell cycle, in the favor of aberrant cell proliferation, is the main mechanism. Thus, these regulatory molecules take role in the induction of apoptosis can also participate in the cell cycle or vice versa. The three molecules mentioned above are very well examples of such regulatory molecules. Also they are all very ideal targets for cancer treatment [17,18].

Survivin (BIRC5), an antiapoptotic protein, has attracted attention of many investigators from the point that it is overexpressed in many types of cancer cells including gastric, lung, colon, breast etc. unlike any other inhibitor of apoptosis proteins (IAPs) [17]. Originally, it was found to prevent cells from apoptosis and was detected highly in the embryonic stage. Subsequently, it was shown to be up-regulated in G2/M phase of the cell cycle and was found to be linked with microtubules and sustained cell survival during G2/M phase. So, survivin acts as an versatile molecule of cell division and apoptosis in cancer [19,20]. Because the highly expression of survivin in many types of cancer is associated with shortage of survival, it is one of the possible targets for cancer treatment in the last years [21-24].

Another such protein participating in the regulation of both apoptosis and cell cycle is MCL-1. The MCL-1 gene (myeloid cell leukemia-1) was discovered incidentally as an induction gene in myeloblastic leukemia cell differentiation about a decade ago and proved to be a member of the emerging Bcl-2 gene family [25]. The antiapoptotic Bcl-2 family member, MCL-1 is normally up- and down-modulated in response to environmental signals and conditions in a normal cell, but is constitutively expressed in cancer where it promotes cell survival and drug resistance [26]. Therefore, MCL-1 has been suggested as a potential new therapeutic target for many types of cancer. In a recent study done by Aicheberger at al., they examined the expression and functional role of MCL-1 in neoplastic mast cells and tried to determine whether MCL-1 could serve as a target in systemic mastocytosis. They found MCL-1 mRNA upregulation in all the patients they have examined and moreover, they have shown that exposure of cells to MCL-1-specific antisense oligonucleotides (ASOs) or MCL-1-specific siRNA resulted in reduced cell survival and increased apoptosis compared with untreated cells [27]. So, MCL-1 is one of the important survival factors for cancer cells and is a potent target of cancer treatment.

The other molecule that was significantly downregulated by docetaxel-ATRA combination in DU-145 cells is also very good example of two-way functioning molecules, lymphotoxin-beta receptor (LTβR). LTβR is a member of the tumor necrosis factor family of signaling receptors that regulate cell survival or death through activation of NF-kappaB. These receptors transmit signals through downstream adaptor proteins called tumor necrosis factor receptor-associated factors (TRAFs) [28]. Mainly, LTβR plays an important role in development and organization of lymphoid tissues acting as a survival factor for cells. But because of the altered control of apoptosis and cell survival in carcinogenesis, it is up-regulated in cancer cells. In some studies, it was clearly shown that by binding LTβR with some specific or oligo-sense antibodies results in decreased tumor growth and increased apoptosis in tumor cells [28-30].

Currently, treatment of HRPC still represents a challenge for clinicians. Since prostate cancer is a disease of elderly men, the wide spectrum toxic side effects of cytotoxic agents used for the treatment are another limitation of daily oncologic practice. Standard first line docetaxel chemotherapy has also some serious side effects in most of the HRPC patients [3,5,6]. Thus, novel and effective approaches may provide an important avenue for the treatment of these groups of patients. We strongly suggest that docetaxel and ATRA combination is a good candidate for this challenging era of daily oncologic practice since the combination of docetaxel and ATRA might allow a reduction in docetaxel doses and by this way may diminish docetaxel adverse effects while maintaining the therapeutic effect in patients with HRPC.

## Competing interests

The authors declare that they have no competing interests.

## Authors' contributions

YK carried out cytotoxicity experiments, and participated in the drafted manuscript, MKG carried out cytotoxicity experiments, EC performed statistical analysis, CE carried out molecular genetic studies, BK participated in the sequence alignment, and drafted manuscript, GG carried out apoptosis experiments, HA carried out apoptosis experiments, and molecular genetic studies, SU participated in design of the study, BK participated in the sequence alignment, UAS participated in the design of the study, RU conceived of the study, and participated in its design and coordination.
